# Study anti-viral drugs for their efficiency against multiple SARS CoV-2 drug targets within molecular docking, molecular quantum similarity, and chemical reactivity indices frameworks

**DOI:** 10.12688/f1000research.146350.1

**Published:** 2024-04-15

**Authors:** Alejandro Morales-Bayuelo, Jesús Sánchez-Márquez, Ricardo Vivas-Reyes, Savaş Kaya

**Affiliations:** 1 Universidad del Sinú, Grupo GENOMA, Cartagena, Bolivar, 36987, Colombia; 2 Departamento de Química-Física, Universidad de Cadiz, Cádiz, Andalusia, 544578, Spain; 3 Group of Quantum and Theoretical Chemistry, Faculty of Exacts and Naturals Sciences, University of Cartagena, Bolívar, Colombia; 4 Faculty of Science Department of Chemistry, Sivas Cumhuriyet University, Sivas, 58140, Turkey

**Keywords:** SARS-CoV-2 virus, COVID-19 treatments, molecular docking, molecular quantum similarity, chemical reactivity indices, Density Functional Theory.

## Abstract

The study focused on drug discovery for COVID-19, emphasizing the challenges posed by the pandemic and the importance of understanding the virus’s biology. The research utilized molecular docking and quantum similarity analyses to explore potential ligands for SARS-CoV-2 RNA-dependent RNA polymerase.

**Docking Results:**

Docking outcomes for various ligands, including Oseltamivir, Prochloraz, Valacyclovir, Baricitinib, Molnupiravir, Penciclovir, Famciclovir, Lamivudine, and Nitazoxanide, were presented. Interactions between ligands and specific residues in the RNA-dependent RNA polymerase were analyzed.

**Reactivity Descriptors:**

Global parameters, such as electronic chemical potential, chemical hardness, global softness, and global electrophilicity, were computed for the ligands. For the local reactivity descriptors, the Fukui Functions were used. Fukui functions, representing electrophilic and nucleophilic sites, were calculated for selected ligands (Valacyclovir and Penciclovir). Nucleophilic character assignments for specific molecular regions were discussed, providing insights into potential charge-donating interactions.

**Results and Discussion:**

Challenges in COVID-19 drug discovery, such as virus mutability, rapid evolution, and resource limitations, were summarized. Progress in vaccine development and the need for ongoing research to address variants and breakthrough cases were emphasized.

**Overlap Operator Analysis:**

Higher MQSM between Lamivudine and Molnupiravir (0.5742) indicates structural and electronic similarity. Lowest MQSM between Oseltamivir and Prochloraz (0.2233) implies structural dissimilarity.

**Coulomb Operator Analysis:**

Higher MQSM between Lamivudine and Molnupiravir (0.9178) suggests both structural and electronic similarity. Lowest MQSM between Baricitinib and Famciclovir (0.6001) indicates greater structural diversity. Measurements above 0.5 in Table 3 suggest electronic similarity, emphasizing the electronic aspects in molecular analysis.

In this sense, it study employed a multi-faceted approach combining molecular docking, quantum similarity analyses, and chemical reactivity assessments to explore potential drug candidates for COVID-19. The findings provide valuable insights into ligand interactions, reactivity patterns, and the challenges associated with drug discovery in the context of the global pandemic.

## 1. Introduction

COVID-19 has caused a significant global public health crisis, with millions of confirmed cases and deaths worldwide. Healthcare systems in various countries faced unprecedented challenges, including shortages of medical supplies, overwhelmed hospitals, and strain on healthcare professionals. While vaccines offered a pathway out of the pandemic, challenges such as vaccine hesitancy, distribution issues, and access disparities in lower-income countries are significant global concerns. The virus responsible for Coronavirus Disease (COVID-19), known as SARS-CoV-2, exhibits varying degrees of symptoms among patients, https://covid19.who.int/. While most individuals undergo mild to moderate symptoms and recover without specific treatment, some progress to severe cases necessitating medical attention. ^1^ Transmission of the virus occurs through microscopic liquid particles expelled from an infected person’s mouth or nose during activities such as coughing, sneezing, speaking, singing, or breathing. These particles, ranging from small aerosols to larger respiratory droplets, can be transmitted through close contact with an infected person or by touching contaminated surfaces and subsequently touching the face. ^2^

As an RdRp (RNA-dependent RNA polymerase), the virus heavily relies on this enzyme for the replication and transcription of its genome. This characteristic makes it an appealing target for the study of its biology and the development of antiviral drugs. ^3^

In the effort to combat COVID-19, multiple drugs are under investigation, and thus far, the FDA has granted approval to only one—remdesivir (Veklury). This antiviral medication is utilized in the treatment of COVID-19 among adults and adolescents aged 12 and above, typically administered intravenously for hospitalized patients. ^4^^,^^5^ Recognizing the significance of comprehending how these drugs stabilize the active site of the receptor structure, this study utilized Molecular Docking, Molecular Quantum Similarity (MQS), and global and local reactivity indices to assess remdesivir and other associated compounds. Examples include Oseltamivir, Prochloraz, Valacyclovir, Baricitinib, Molnupiravir, Valacyclovir, Penciclovir, Famciclovir, Lamivudine, and Nitazoxanide.

The MQS concept, introduced by Carbo-Dorca and colleagues, analyzes molecular similarities among different compounds. In this research, Density Functional Theory (DFT) was employed to bridge the gap between Molecular Quantum and Quantum Chemistry, combining molecular docking with chemical reactivity indices. ^5^ This approach provided valuable insights into potential alternative treatments for COVID-19 and shed light on the interactions between approved drugs like remdesivir and other potential ligands.

Molecular docking plays a crucial role in drug discovery for COVID-19 by facilitating the identification and design of potential therapeutic compounds. Here are some key aspects highlighting the importance of molecular docking in the context of COVID-19 drug discovery: Target Identification and Validation: Molecular docking helps identify potential drug targets within the SARS-CoV-2 virus, such as viral proteins critical for its replication and survival. It aids in validating the chosen targets by predicting the binding affinity and interaction patterns of small molecules with these targets.

Docking simulations provide insights into the binding mechanisms between potential drug candidates and viral proteins. This information is crucial for understanding how a drug may interfere with the virus’s life cycle. Molecular docking predicts the binding affinity of a ligand to a target protein. Compounds with high binding affinity are more likely to have therapeutic effects, making them promising candidates for further experimental validation. Rational Drug Design: Docking studies guide rational drug design by providing a structural basis for modifying existing drugs or designing new compounds that specifically target essential viral proteins. Antiviral Drug Development: The identification of potential drug candidates through molecular docking contributes to the development of antiviral drugs targeting specific proteins crucial for the virus’s replication or entry into host cells. Cost and Time Efficiency: Computational approaches like molecular docking significantly reduce the time and costs associated with drug discovery by prioritizing the most promising compounds for experimental validation.

## 2. Methods

### 2.1 System preparation

In the docking experiment, the receptor structure was derived following specific protocols based on the crystal structure of SARS-CoV-2 RNA-dependent RNA polymerase with PDB code 6m71. Adjustments to the structure were made using the protein preparation wizard module from the Schrödinger suite 2017-1. These adjustments included:

i) Optimization of the hydrogen bond (H-bond) network and refinement of the protein structure.
ii) Determination of protonation states at physiological pH using the PropKa utility.
iii) Execution of restrained molecular minimization through the Impact Refinement (Impref) module, with heavy atoms constrained to a low root-mean-square deviation (RMSD) from the initial coordinates (chrome-extension://efaidnbmnnnibpcajpcglclefindmkaj/ https://www.modekeji.cn/wp-content/uploads/2019/08/gli55_user_manual.pdf). ^6^^–^^8^

Conversely, the molecular structures of the compounds were constructed using the Maestro Editor (Maestro, version 11.1, Schrödinger, LLC). Subsequently, 3D conformations were generated using the LigPrep module, and ionization/tautomeric states were predicted under physiological pH conditions using Epik. Finally, energy minimization was performed with the Macro model using the OPLS2005 force field.

### 2.2 Molecular docking

Glide ( https://newsite.schrodinger.com/platform/products/glide/) ^9^^,^^10^ with default parameters and Standard Precision (SP) model was used for docking investigations. The docking grid was created using default settings, with the co-crystallized ligand in the center. For the van der Waals radii of the nonpolar protein atoms, a scaling factor of 0.8 was applied to facilitate the binding of larger ligands. Extra precision (XP) was also utilized to expand alternate receptor conformations appropriate for binding ligands with unusual orientations via induced fit docking (IFD); this method allows the protein to undergo side-chain, backbone, or both movements upon ligand docking. All results were redocking and RMSD were performed. The binding pocket of the RdRp—GLY616, TRP617, ASP618, TYR619, LEU758, SER759, ASP760, ASP761, ALA762, LYS621, TYS799, TRP800, GLU811, PHE812, CIS813, and/or SER814—was found using Glide. ^9^^,^^10^

The docking process involves four precise steps, relying on Glide’s scoring function and Prime’s advanced conformational refinement to ensure accuracy:

(i) Initial docking using Glide is executed on the rigid receptor to generate a set of poses.
(ii) The side-chain prediction module of the Prime module is employed to sample the protein, followed by structural minimization for each pose of the protein/ligand complex.
(iii) Redocking of the ligand into the low-energy induced-fit structures from the previous step is conducted using Glide’s default parameters (without vdW scaling).
(iv) The binding energy (IFDScore) is estimated, considering the docking energy (GScore), receptor strain, and solvation terms (Prime energy).

To further assess the interactions of the ligands in the active site, the extent of residue movement induced by the IFD computation is considered. For both the most and least active ligands, all poses are compared within the molecular set. Molecular dynamics calculations over 30ns are employed to predict the best poses and analyze their stabilization in the active site.

## 3. Quantum Similarity Analyses

### 3.1 Molecular Quantum Similarity Measure

A Molecular Quantum Similarity Measure (MQSM) amid two A and B systems, known as Z _AB_, compares two molecules that may be created using their respective Density Functions (DFs).

Both DFs can be multiplied and integrated in terms of their electronic coordinates, which are then weighted using a predetermined positive operator Ω( *r*_1_, *r*_2_) ^11^^–^^13^:

$$Z_{\mathrm{AB}}=\left\langle \mathrm{\rho A}\right|\Omega \left|\mathrm{\rho B}\right\rangle =\int \int \mathrm{\rho A}\left(r_{1}\right)\Omega \left(r_{1},r_{2}\right)\mathrm{\rho B}\left(r_{2}\right){\mathrm{dr}}_{1}{\mathrm{dr}}_{2}$$ (1)

The operator used in Equation 1 plays a crucial role in determining the information being compared and serves as the measure of similarity between the two systems. For example, when the operator chosen is the Dirac delta function, it proves to be an efficient approach for functions with high peak values, like the electronic density. Moreover, it provides a similar mathematical abstraction as a charge or point mass, i.e., Ω( *r*_1_, *r*_2_) = δ( *r*_1_ - *r*_2_). One of the first similarity metrics employed is the overlapping MQSM; another widely used alternative is the Coulomb operator, i.e., Ω( *r*_1_, *r*_2_) = |‌‌ *r*_1_ - *r*_2_|‌‌ ^−1^, resulting in a Coulombic MQSM. Even if the two molecules are equivalent, a similarity measure can be employed for any two molecular systems; this measurement is known as a self-similarity measure ( *Z*_*AA*_ for the case of molecule *A*). ^12^

For a given group of N molecules, we can derive a measure of similarity for each molecule concerning the others in the group, including itself. These similarity measurements can then be used to construct a symmetric matrix. The *i*-th column of this matrix represents a compilation of all similarity measurements between the *i*-th molecule and every constituent in the group, including itself. Consequently, each vector (matrix column) serves as a discrete N-dimensional representation of the *i*-th structure. These vector sets can be demonstrated as a set of chemical descriptors. However, this set of similarity matrix columns goes beyond merely representing another set of molecular descriptors, as commonly done for theoretical molecule description; each descriptor possesses unique properties. ^12^^–^^20^

i) It is universal, deriving from any collection of molecules and any individual molecule within that group.
ii) It is impartial, as there are no other possibilities available in the construction process than those dictated by the density functions and similarity measurements involved.

### 3.2 Carbó’s similarity index

$$C_{\mathrm{IJ}}\left(\Omega \right)=z_{\mathrm{IJ}}\left(\Omega \right){\left[z_{\mathrm{II}}\left(\Omega \right)z_{\mathrm{JJ}}\left(\Omega \right)\right]}^{-1/2}$$ (2)

Carbó’s similarity index between two molecules *I* and *J* are constructed from Equation 2. Because this index is also known as the cosine similarity index, it corresponds to the cosine of the angle included by the density functions involved when considered as vectors. For any pair of compared molecules, this Carbo QSI has a value between 0 and 1, depending on the similarity between the two molecules (when *I* = *J*, the index approaches 1). ^13^^–^^28^

### 3.3 The quantum similarity using the Euclidean distance

Taking into account the similarity of equation 3:

$$\;D_{\mathrm{IJ}}\left(k,x,\Omega \right)={\left[k\left(z_{\mathrm{II}}\left(\Omega \right)+z_{\mathrm{JJ}}\left(\Omega \right)\right)/2–{\mathrm{xz}}_{\mathrm{IJ}}\left(\Omega \right)\right]}^{1/2},x\left[0,k\right]$$ (3)

It is simplified to the so-called Euclidean distance index when *k* = *x* = 2. Index 3 of the form can also be defined as follows:

$$D_{\mathrm{IJ}}\left(\infty ,\Omega \right)=\max\left(z_{\mathrm{II}}\left(\Omega \right),z_{\mathrm{JJ}}\left(\Omega \right)\right)\;$$ (4)

This Equation 4 forms the distance index of infinite order. ^18^^–^^30^

### 3.4 MQSM overlap considering the Equation 2

The distribution of Dirac’s delta, Ω ( *r*_1_, *r*_2_) = δ ( *r*_1_, *r*_2_), is the most typical and intuitive choice for such a positively defined operator. These selections transform the broad definition of MQSM to compute the overlap MQSM that obtains measurements of the volume by both electronic density functions when they are superimposed. ^17^^–^^20^

$$z_{\mathrm{IJ}}\left(\Omega \right)=\int \int \rho_{I}\left(r_{1}\right)\delta \left(r_{1}-r_{2}\right)\rho_{J}\left(r_{2}\right){\mathrm{dr}}_{1}{\mathrm{dr}}_{2}=\int \rho_{I}\left(r\right)\rho_{J}\left(r\right)\mathrm{dr}$$ (5)

The Dirac delta function is derived instinctively from the physical definition, and it is computationally compliant. The MQSM calculates the degree of overlap between molecular comparisons using information about the electron concentration in the molecule. ^16^^–^^21^

### 3.5 MQS Coulomb considering the Equation 5

When the operator (Ω) is replaced with the Coulomb operator, Ω (r _1_, r _2_)= $\frac{1}{│r_{1}-r_{2}│}$ , the coulomb MQS is generated, which defines the electrostatically repellent coulomb energy between two charge densities ^20^^,^^21^:

$$Z_{\mathrm{IJ}}\left(\Omega \right)=\int \int \rho_{I}\left(r_{1}\right)\frac{1}{r_{1}-r_{2}}\rho_{J}\left(r_{2}\right){\mathrm{dr}}_{1}{\mathrm{dr}}_{2}$$ (6)

The coulomb operator affects the overlap density functions. When considering molecular density functions as an electron distribution in space, this equation is simply an extension of the coulomb operator for the distribution of continuous charge, thus can be used as electrostatic potential descriptors in some instances. This operator is correlated to electrostatic interactions and obtains the measurement of electrostatic repulsion between electronic distributions. ^30^^–^^37^

### 3.6 Euclidean distance index considering the Equation 3

Another major transformation that can be expressed in terms of the classical distance is:

dab=∑j=1pΔxjk1k (7)

Here $\Delta x_{j}=x_{\mathrm{aj}}-x_{\mathrm{bj}}$ is the distance between *a* and *b*, and *k* = 2 is the definition of distance, respectively. Subsequently, the Euclidean distance between A and B as two quantum objects are defined by ^17^^–^^21^:

$$d_{\mathrm{ab}}=\sqrt{{\left(x_{a}-x_{b}\right)}^{2}}.$$ (8)

Occasionally it is written as: $D_{\mathrm{AB}}=\sqrt{Z_{\mathrm{AA}}+Z_{\mathrm{BB}}+{\mathrm{ZZ}}_{\mathrm{AB}}}$ , where *D*_*AB*_ has values in the range of [0,∞) but for earlier circumstances where the compared items are identical, it converges to zero between them. ^17^^–^^21^:

$$D_{\mathrm{AB}}=0$$ (9)

The norm of the differences in the density functions of the compared objects can be used to interpret this index geometrically. The distance or dissimilarity index can be used to define the Euclidean distance index, which can also be represented as ^21^^–^^25^:

$$D_{\mathrm{AB}}=∥\rho_{A}-\rho_{B}∥=\sqrt{{\left(\rho_{A}-\rho_{B}\right)}^{2}}$$ (10)

### 3.7 Alignment method: Topo-Geometrical Superposition Algorithm (TGSA)

In this investigation, the TGSA (Typical Geometry Superposition Algorithm) approach was utilized for data alignment. Devised by Gironés, TGSA operates under the assumption that the optimal way to align molecules involves superimposing them onto a shared skeleton, considering solely the atomic types and interatomic bonding interactions based on the atomic number coordination. ^23^^–^^28^

The program initiates by scrutinizing pairs of atoms in the molecules, aligning their common substructure for a group of molecules using topological and geometrical considerations. Notably, the superposition achieved is distinctive and unaffected by the choice of similarity measure. ^28^^–^^34^

Initially, the program organizes molecular coordination into bases based on the reduction of atomic numbers, defining a path for the number of hydrogens in the molecule (excluding hydrogen atoms for computational efficiency). Subsequently, atomic pairs are delineated, specifying the involved atoms and their respective distances. ^34^^–^^37^ Translocations are identified through changes in the conformations’ spine caused by substitutions in the molecules. ^23^ Bones not aligning with the skeletons are eliminated during this process.

The program then assembles atomic triads by incorporating three atoms from the compared pairs, forming a triangle in the plane representing the chemical box’s efficacy. ^23^^,^^24^ The triangles generated for two molecules are compared using their respective interatomic and translational distances. Triads meeting the classification criteria are retained, superimposed, and dictate the molecular alignment result. ^23^

It’s worth noting that since TGSA characterizes molecules as rigid structures without flexibility (no vibration or rotation in bond distances and angles), it may not yield optimal results for diverse molecular structures due to the restricted alignment with the common recognition skeleton. Nevertheless, this method consistently aligns with chemical intuition and is favored for its accessibility and lower computational requirements. ^26^^–^^37^

## 4. Chemical Reactivity Analysis

Studies in the field have demonstrated an established link between quantum similarity and descriptors of chemical reactivity. ^38^^,^^39^ Both quantum similarity and DFT employ the density function as a key element in examining similarity indices. Specifically, the Coulomb index can be associated with electronic aspects that influence chemical reactivity. To determine global reactivity indices such as chemical potential (μ), ^27^ hardness (ɳ), ^38^ and electrophilicity (ω), ^39^^,^^40^ Frontier Molecular Orbitals (FMO) and the energy gap will be utilized for computation. These indices ( Equations 11- 13) offer valuable insights into the stability of systems, with chemical potential gauging electron tendency to depart from the equilibrium system, ^41^ while chemical hardness assesses a chemical species’ resistance to altering its electronic configuration.

$$\mu \approx \frac{E_{\text{LUMO}+E_{\text{HOMO}}}}{2}$$ (11)

$$\eta \approx E_{\text{LUMO}}-E_{\text{HOMO}}$$ (12)

The mathematical definition of the electrophilicity index (ω) is related to the stabilization energy of a system when it becomes saturated by electrons from the external environment. ^39^^,^^40^:

$$\omega =\frac{\mu^{2}}{2\eta }$$ (13)

In this research, the local reactivity descriptors under consideration were the Fukui functions. The Equations ^42^^,^^43^ illustrate the system’s electronic density response to variations in the global charge, representing the derivative of the electronic density concerning the electron count under a consistent external field.

$$f^{+}\left(\vec{r}\right)\approx {\left|\text{LUMO}\left(\vec{r}\right)\right|}^{2}$$ (14)

$$f^{-}\left(\vec{r}\right)\approx {\left|\text{HOMO}\left(\vec{r}\right)\right|}^{2}$$ (15)

The terms $f^{+}\left(\vec{r}\right)$ and $f^{-}\left(\vec{r}\right)$ have been employed to denote nucleophilic and electrophilic attacks, respectively. ^31^^–^^33^ This approach utilizes both global and local reactivity descriptors to examine quantum similarity within the molecular set. All computations were conducted using the B3LYP method ^44^ with the 6-311XXG(d,p) basis set, ^45^ which involves an improvement to the 6-311G(d) basis set. This enhancement allows for calculations of electronegativity, hardness, reactivity indices, and frontier molecular orbitals at a quality level comparable to much larger basis sets like Aug-cc-pVQZ and Aug-cc-pV5Z. The Gaussian 16 package ^46^ was employed in conjunction with this method/basis set combination.

## 5. Results and Discussion

### 5.1 Molecular docking outcomes for the ligands

Drug discovery for COVID-19 presents several challenges, and researchers worldwide have been working diligently to address these issues. Some of the key challenges include: *Virus Mutability*: SARS-CoV-2, the virus responsible for COVID-19, can mutate, leading to the emergence of new variants. This mutability poses a challenge in developing drugs that can effectively target different strains of the virus. *Rapid Evolution of the Pandemic*: The rapid spread of the virus and the urgent need for effective treatments make it challenging to follow traditional drug development timelines. Accelerated timelines can compromise thorough testing and validation processes. *Lack of Pre-existing Therapies*: Unlike some other infectious diseases, there were no pre-existing drugs specifically designed to target SARS-CoV-2. Developing new drugs from scratch is a time-consuming process.

**Figure 1.**
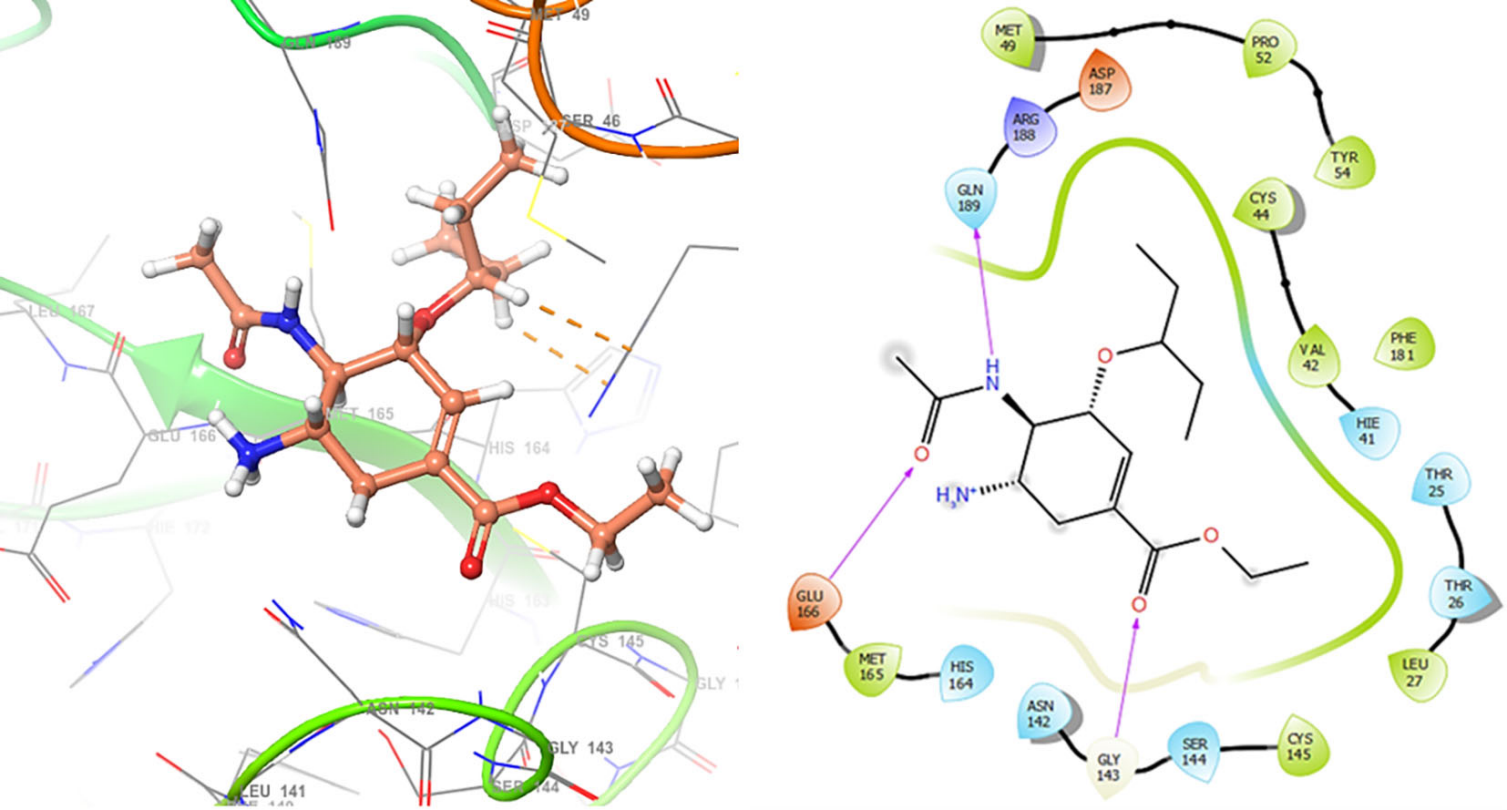
Docking results for Oseltamivir.

**Figure 2.**
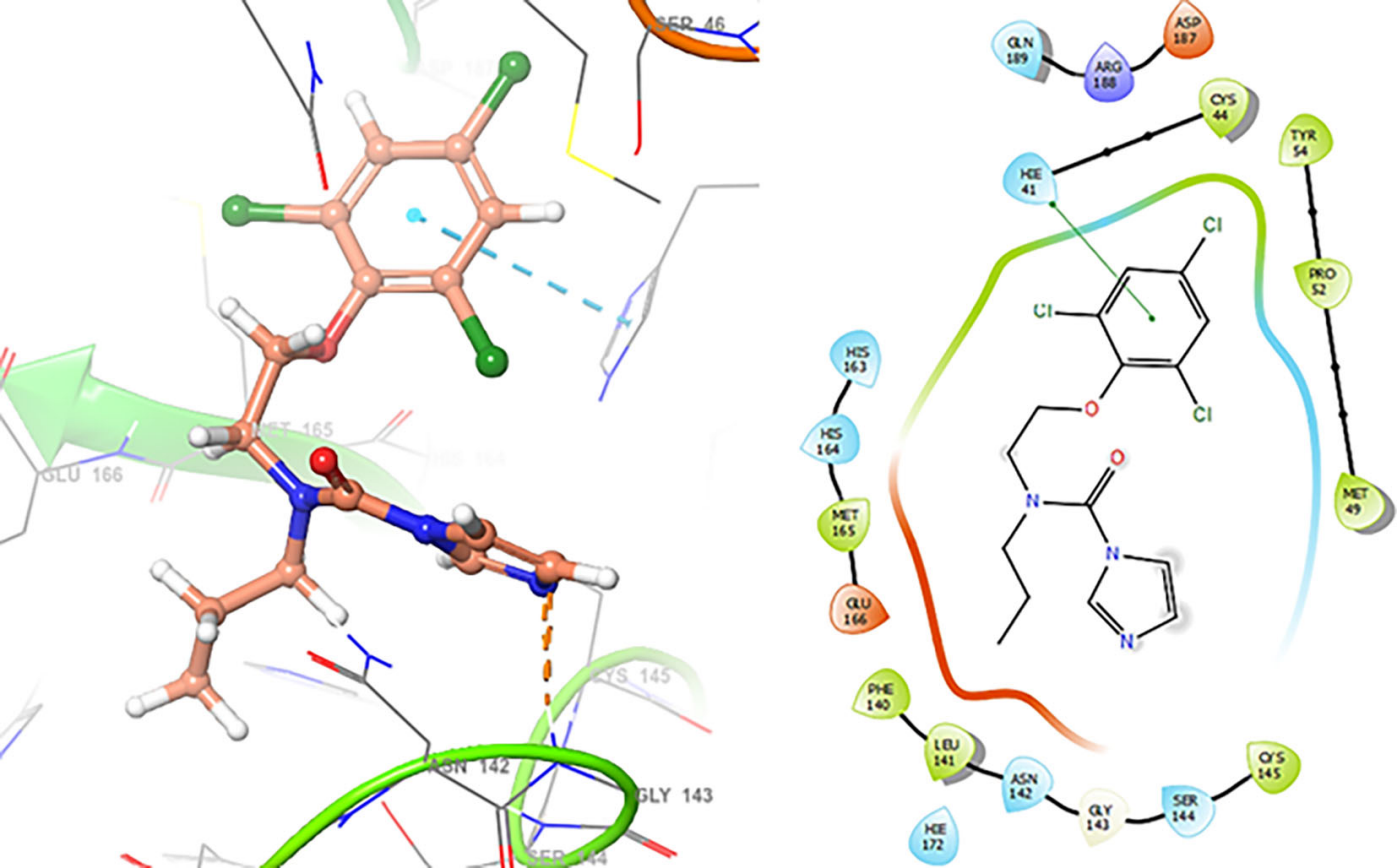
Docking results for Prochloraz.

*Complexity of the Virus Life Cycle*: Understanding the intricate details of the virus’s life cycle and the host-pathogen interactions is essential for developing targeted therapies. This complexity requires a deep understanding of virology and immunology. *Drug Safety*: Ensuring the safety of potential treatments is crucial. Some drugs may show promise in early stages but could have adverse effects or interactions with other medications, requiring extensive testing for safety. *Drug Delivery Challenges*: Designing effective drug delivery systems to ensure that the drug reaches the target tissues in sufficient concentrations is a significant challenge. This is especially important for antiviral drugs targeting the respiratory system. *Antibody Resistance*: The virus may develop resistance to certain antiviral drugs or antibodies over time. This highlights the need for ongoing research to identify multiple targets for drug development and combination therapies. *Global Collaboration*: International collaboration is crucial for sharing data, resources, and expertise. However, coordinating efforts across borders and overcoming logistical and political challenges can be complex. *Vaccine Success and Impact*: The success and widespread distribution of COVID-19 vaccines have been crucial in controlling the pandemic. However, ongoing research is needed to address vaccine effectiveness against new variants and to develop treatments for breakthrough cases. *Resource Limitations*: Drug discovery requires significant financial and human resources. The COVID-19 pandemic has strained healthcare systems globally, and prioritizing and allocating resources for research and development can be challenging. Despite these challenges, the scientific community has made remarkable progress in a short time, developing vaccines and exploring various therapeutic approaches. Continuous research and collaboration will be essential for addressing the evolving nature of the pandemic and improving our ability to manage and treat COVID-19. For these reasons, in this study are obtained new insights for a serie of ligands, based on the crystal structure of SARS-CoV-2 RNA-dependent RNA polymerase with PDB code 6m71. Please, see Figures 1 and 2.

In Figure 1 are shows the docking outcomes for Oseltamivir. The main interaction is with the residues GLU166 (-H, 1.323 Å), GLN189 (-H, 1.185 Å) and GLY143 (-H, 1.054 Å). However, in Figure 2 for Prochloraz has a stacking (also called π–π stacking, (1.213 Å)) with the residues HIE41.

**Figure 3.**
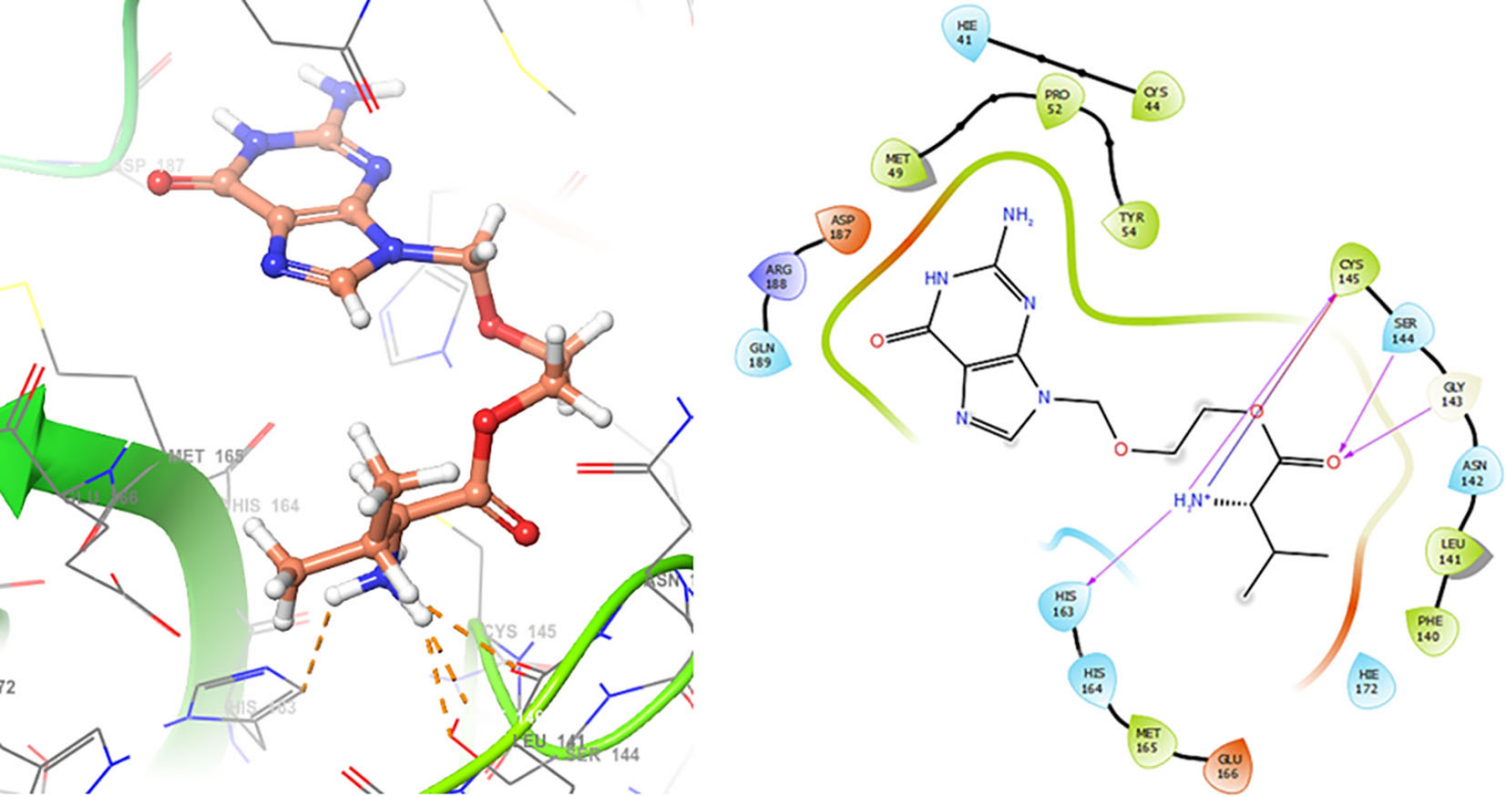
Docking results for Valacyclovir.

The Figure 3 shows the docking results for Valacyclovir, this ligand shows interactions with the residues CYS145 (-H, 1.452 Å), SER144 (-H, 1.114 Å), GLY143 (-H, 1.156 Å) and HIS163 (-H, 1.254 Å).

**Figure 4.**
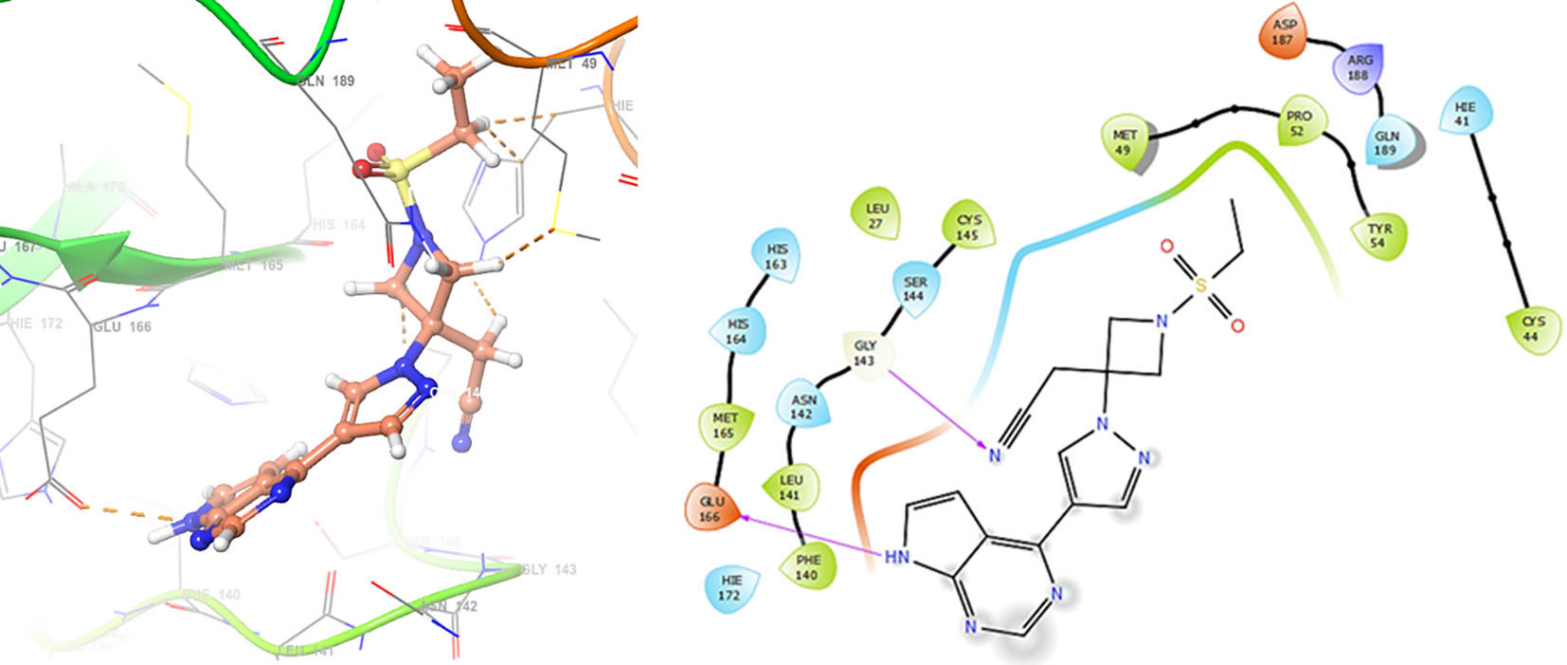
Docking results for Baricitinib.

**Figure 5.**
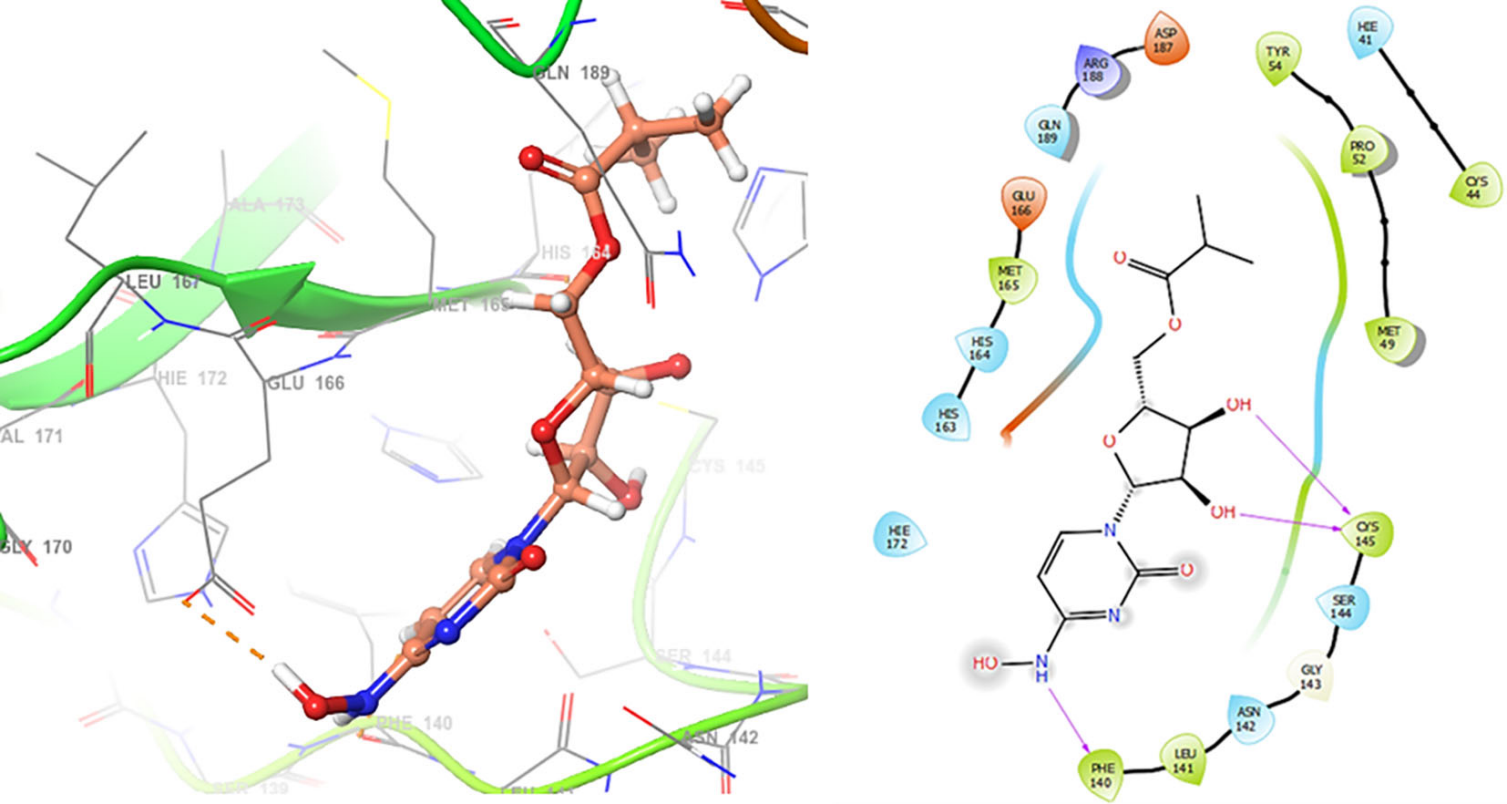
Docking results for Molnupiravir.

In Figure 4 show the interaction for Baricitinib, this Figure shows interactions with the residues GLU166 (-H, 1.568 Å), GLY143 (-H, 1.365 Å). Unlike, in the Figure 5 we can see interactions for Molnupiravir with the residue CYS145 (-H, 1.248 Å and 1.238 Å).

**Figure 6.**
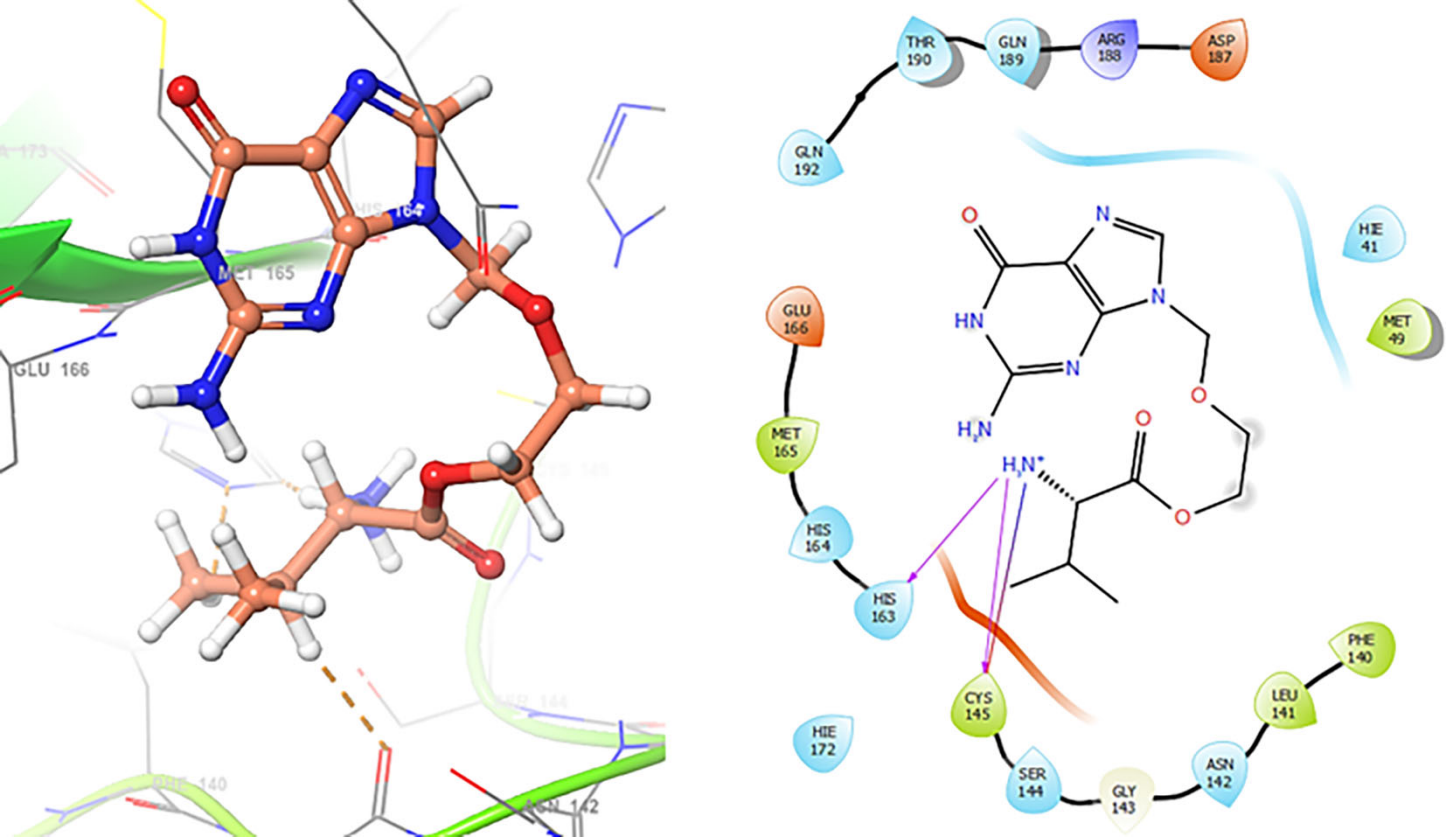
Docking results for Valacyclovir.

**Figure 7.**
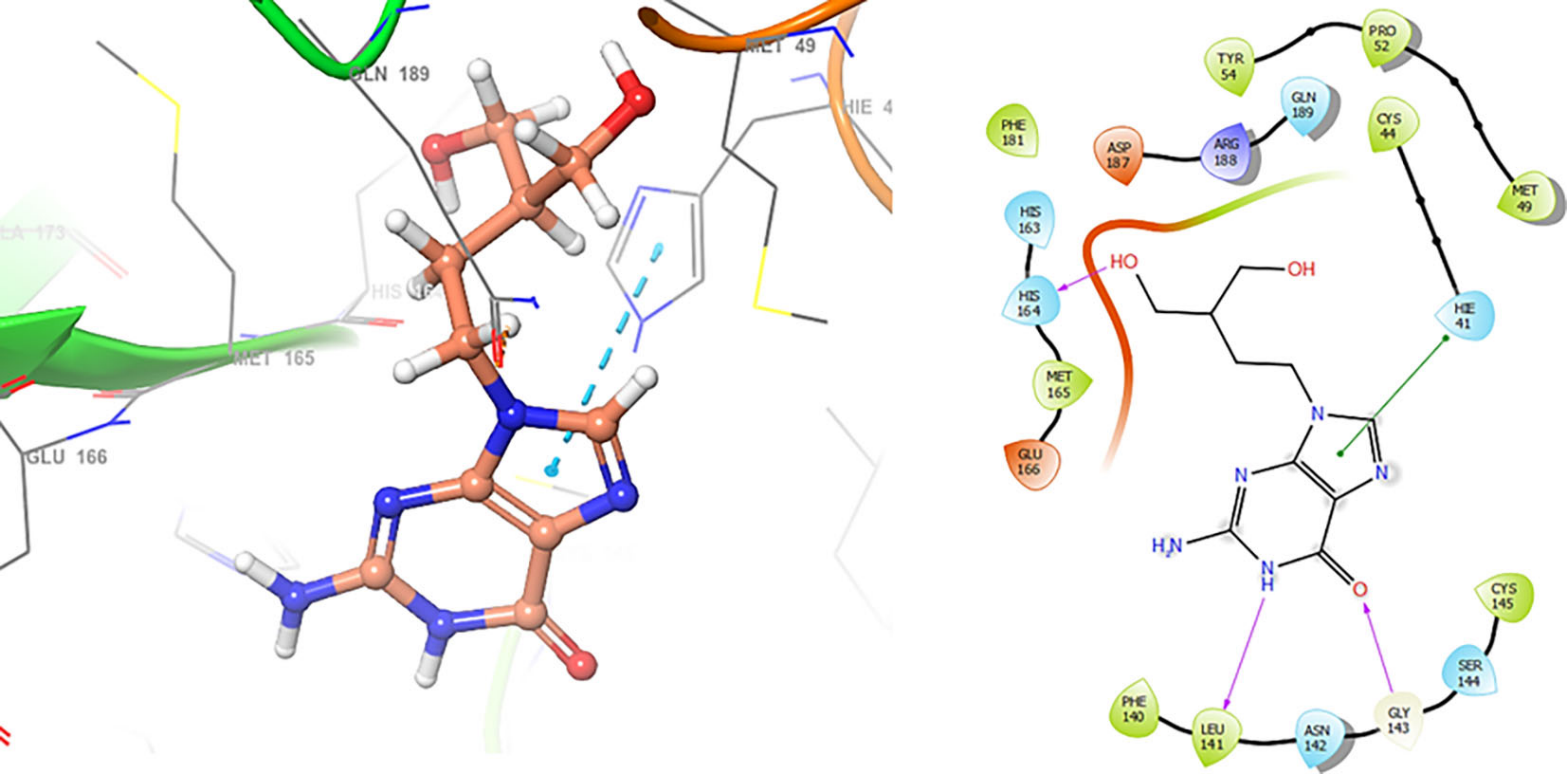
Docking results for Penciclovir pose 1.

In Figure 6, shows the interactions for Valacyclovir with the residue HIS163 (-H, 1.156 Å) and CYS145 (-H, 1.256 Å). The Figure 7, shows the interactions for Penciclovir with the residues HIS164 (-H, 1.005 Å) and π-π interactions with the residues HIE41 (-H, 1.269 Å)

**Figure 8.**
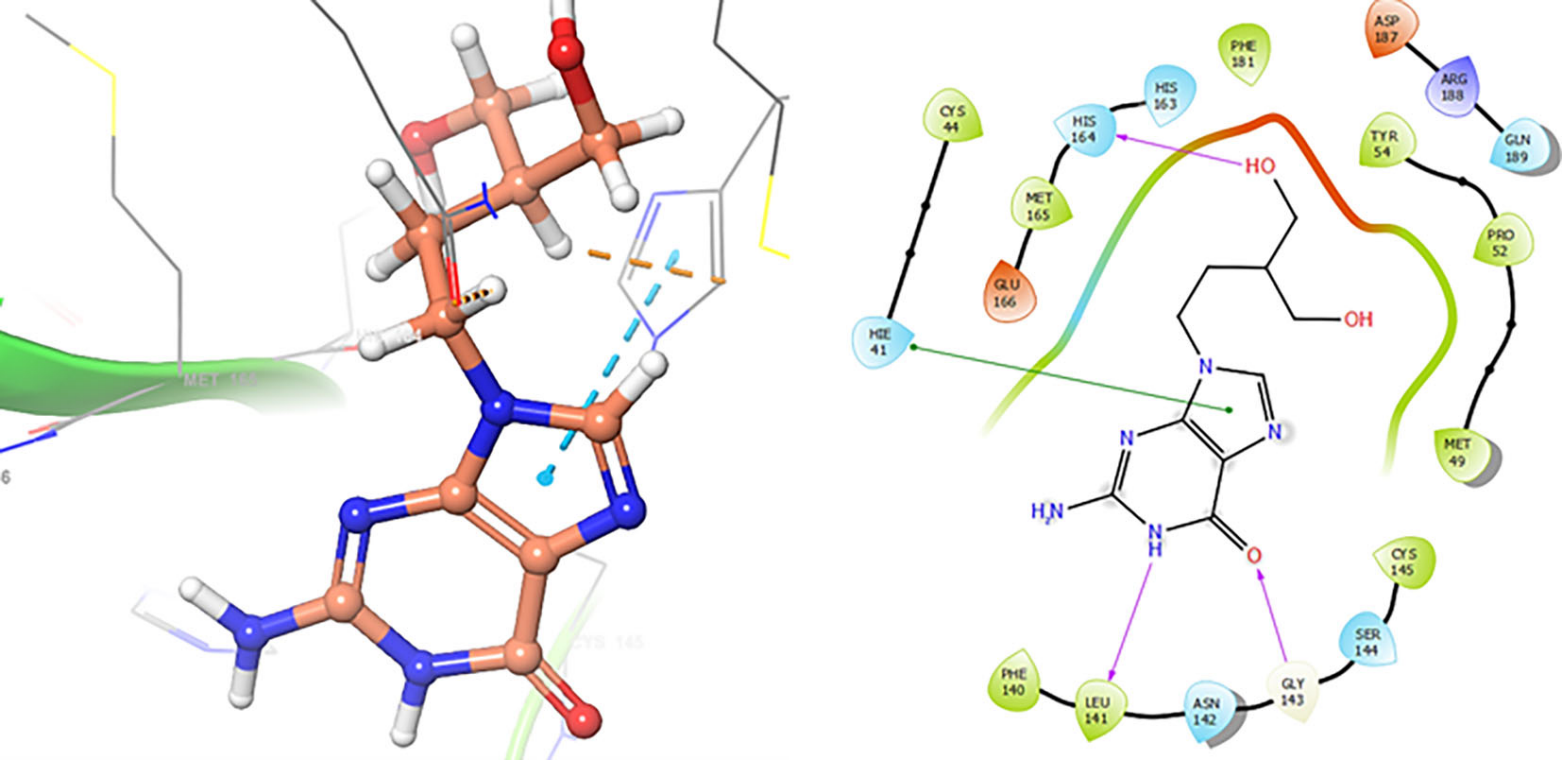
Docking results for Penciclovir pose 2.

**Figure 9.**
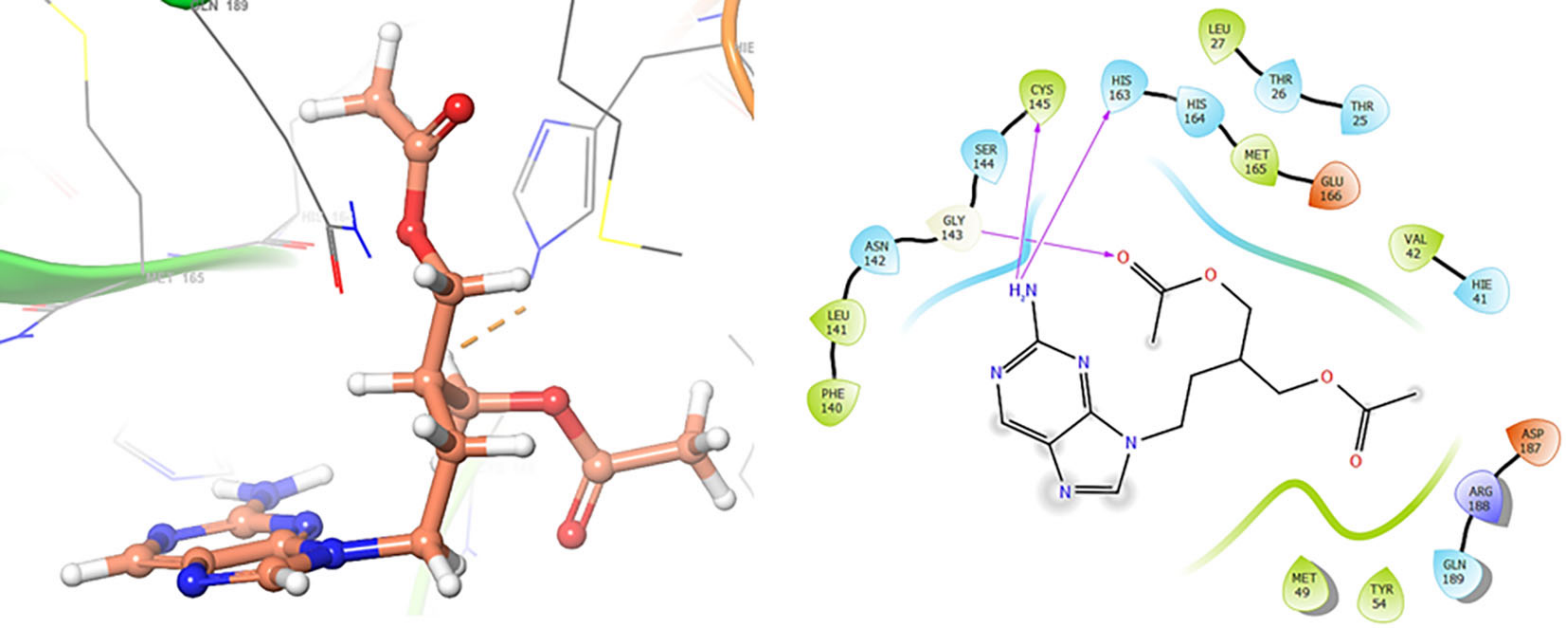
Docking results for Famciclovir.

In the Figures 8 and 9, shows the interactions with the residues HIE41 (-H, 1.567 Å) for Penciclovir and with the residues GLY143 (-H, 1.456 Å), CYS145 (-H, 1.436 Å) and HIS163 (-H, 1.485 Å).

**Figure 10.**
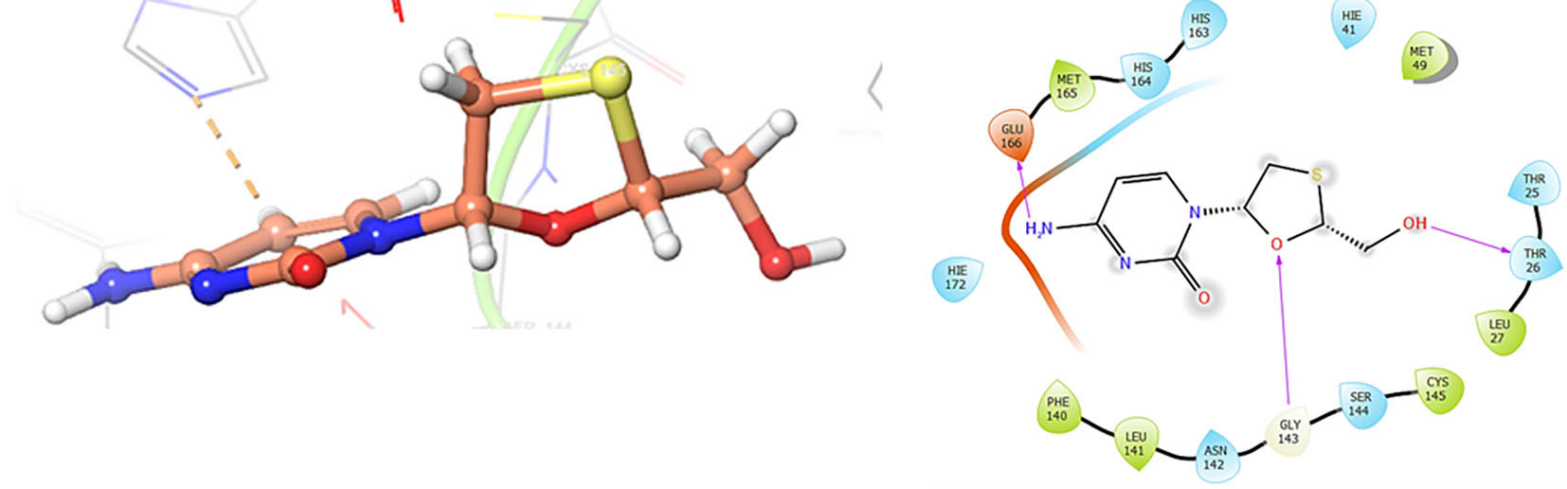
Docking results for Lamivudine.

**Figure 11.**
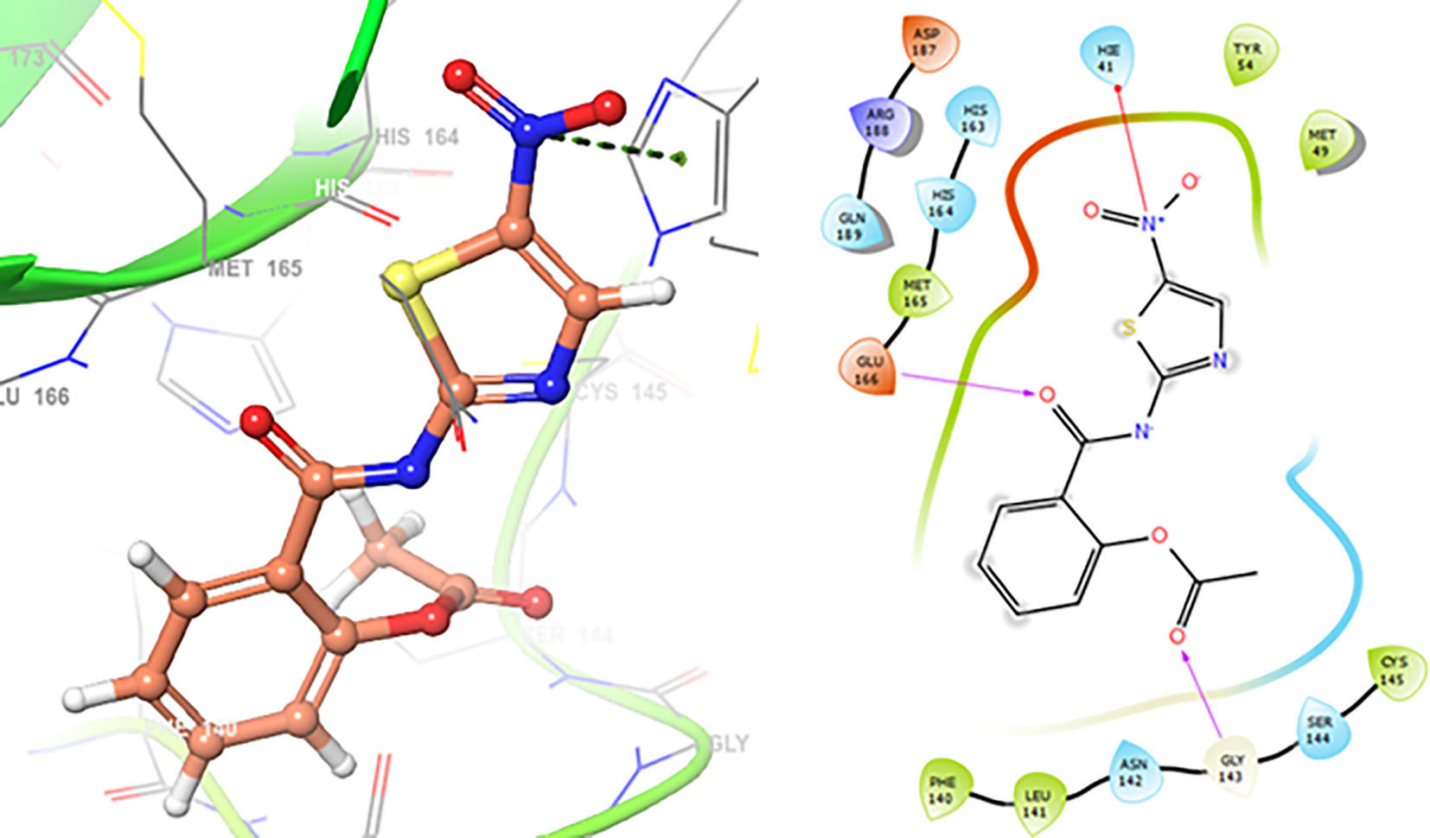
Docking results for Nitazoxanide.

Finally, in the Figures 10 and 11 we can see the interactions with the residues GLU166 (-H, 1.054 Å) and GLY143 (-H, 1.158 Å) for Lamivudine and with the residues GLU166 (-H, 1.254 Å), HIE41 (-H, 1.266 Å) and GLY143 (-H, 1.354 Å) for Nitazoxanide.

### 5.2 Molecular quantum similarity analysis

MQSM helps in the identification of molecules with similar quantum properties, which can be useful for finding potential drug candidates. Similar quantum properties suggest similar chemical behaviours. MQSM involves the calculation of quantum descriptors, which are numerical representations of molecular properties derived from quantum mechanical calculations. These descriptors may include electronic density, electron localization function, and others. Various similarity measures, such as Carbo’s similarity index or Euclidean distance, are employed to quantify the degree of similarity between quantum descriptors of different molecules MQSM can aid in lead optimization by identifying compounds with quantum properties like known drug candidates. This assists in designing molecules with improved pharmacological profiles.

The MQSM can be used to understand the interactions between ligands and their target receptors at a quantum level. This is crucial for rational drug design. It is often used in conjunction with molecular docking studies. While docking predicts the binding affinity and geometry of ligands with target proteins, MQSM provides insights into the quantum properties that influence these interactions.

MQSM offers a detailed and atomistic understanding of molecular properties, allowing for a more nuanced analysis of chemical similarity. Unlike traditional structural similarity measures, MQSM considers the quantum properties of molecules, providing a more comprehensive comparison.

In the context of antiviral drug discovery, MQSM can be employed to identify molecules with similar quantum properties to known antiviral drugs, aiding in the search for new therapeutics. Therefore, Molecular Quantum Similarity analysis is a valuable tool in drug discovery that leverages quantum mechanical principles to assess the similarity of molecular properties. This approach contributes to the rational design of novel drugs and the optimization of lead compounds for improved pharmacological profiles.

In Tables 1 and 2. the higher MQSM is between the compounds Lamivudine and Molnupiravir using the Overlap operator 0,5742 with a euclidean distance of 4,2364, see Table 2. The lowest MQSM is between the compounds Oseltamivir and Prochloraz (0,2233) with a eclidean distance of 5,5841 (see Table 2). These measurements are below 0.5, according to the range of carbon indices (0.1]. So we can say that structurally they are quite different.

**Table 1.** Molecular quantum similarity using the Overlap operator.

O_Hab	Bari	Famc	Fosc	Lami	Moln	Nita	Osel	Paxl	Penc	Proc	Vala
**Bari**	1.0000										
**Famc**	0.3343	1.0000									
**Fosc**	0.3049	0.3411	1.0000								
**Lami**	0.3445	0.4377	0.4803	1.0000							
**Moln**	0.2872	0.3861	0.4172	0.5742	1.0000						
**Nita**	0.4494	0.2946	0.2954	0.5384	0.3904	1.0000					
**Osel**	0.2608	0.3090	0.4264	0.3855	0.3108	0.3971	1.0000				
**Paxl**	0.4279	0.2604	0.3040	0.4816	0.4117	0.2428	0.3188	1.0000			
**Penc**	0.4140	0.4863	0.4047	0.5429	0.4426	0.3776	0.2020	0.4074	1.0000		
**Proc**	0.3507	0.3356	0.4290	0.4131	0.3169	0.4192	0.2233	0.2785	0.4342	1.0000	
**Vala**	0.3660	0.4319	0.4117	0.4796	0.4115	0.2982	0.3152	0.3573	0.6339	0.3149	1.0000

**Table 2.** Eucliean diastance using the Overlap operator.

O_Dab	Bari	Famc	Fosc	Lami	Moln	Nita	Osel	Paxl	Penc	Proc	Vala
**Bari**	0.0000										
**Famc**	5.4654	0.0000									
**Fosc**	4.8543	4.7442	0.0000								
**Lami**	4.9726	4.6222	3.6619	0.0000							
**Moln**	5.8372	5.4225	4.7179	4.2364	0.0000						
**Nita**	4.8681	5.5139	4.7364	4.0802	5.3032	0.0000					
**Osel**	5.6683	5.4850	4.3458	4.7238	5.6619	5.0123	0.0000				
**Paxl**	6.2742	7.0642	6.4218	5.8371	6.4570	7.0562	6.7427	0.0000			
**Penc**	4.8802	4.5767	4.1761	3.8947	4.9488	4.9105	5.5908	6.2391	0.0000		
**Proc**	5.1939	5.2582	4.1763	4.4787	5.5227	4.8022	5.5841	6.8370	4.5857	0.0000	
**Vala**	5.3749	5.0917	4.5639	4.4999	5.3457	5.5444	5.5041	6.6462	3.9109	5.3850	0.0000

In Tables 3 and 4 the higher MQSM using the Coulomb operator is between the compounds Lamivudine and Molnupiravir (0,9178) with a euclidean distance of 22,5461, see Table 4. However, the lowest MQSM is between the compounds Baricitinib and Farmciclovir (0,6001) with a euclidean distance of 44,3298. The comparison between the compounds Lamivudine and Molnupiravir has the higher values in both case using the Overlap and Coulomb operators. Unlike to the MQSM using the overlap operator, the measurements of the Table 3 are above 0.5, according to the range of carbon indices (0.1]. Therefore, we can say that although structurally they are not so similar, electronically they are. Because the electronic similarity index is higher with respect to the overlap, we have analyzed in depth the electronic properties of the analyzed ligands.

**Table 3.** Molecular quantum similarity using the Coulomb operator.

C_Hab	Bari	Famc	Fosc	Lami	Moln	Nita	Osel	Paxl	Penc	Proc	Vala
**Bari**	1.0000										
**Famc**	0.6001	1.0000									
**Fosc**	0.6450	0.6272	1.0000								
**Lami**	0.6471	0.8474	0.8663	1.0000							
**Moln**	0.6479	0.8528	0.6673	0.9178	1.0000						
**Nita**	0.9087	0.7880	0.5885	0.8840	0.8421	1.0000					
**Osel**	0.7881	0.6362	0.7776	0.8339	0.6477	0.8304	1.0000				
**Paxl**	0.8971	0.6692	0.7660	0.8952	0.8363	0.6483	0.8077	1.0000			
**Penc**	0.6739	0.8017	0.7833	0.8386	0.8482	0.7665	0.7486	0.8472	1.0000		
**Proc**	0.7672	0.7665	0.8041	0.7526	0.7212	0.7549	0.7737	0.8437	0.8472	1.0000	
**Vala**	0.6393	0.7709	0.7138	0.7717	0.8528	0.7547	0.7056	0.8213	0.9060	0.8064	1.0000

**Table 4.** Eucliean diastance using the Coulomb operator.

C_Dab	Bari	Famc	Fosc	Lami	Moln	Nita	Osel	Paxl	Penc	Proc	Vala
**Bari**	0.0000										
**Famc**	44.3298	0.0000									
**Fosc**	40.3077	38.3534	0.0000								
**Lami**	38.8901	26.0049	20.1286	0.0000							
**Moln**	42.5088	26.8417	39.3225	22.5461	0.0000						
**Nita**	21.4107	30.1434	35.2862	20.4464	27.2328	0.0000					
**Osel**	32.5547	41.4439	34.7428	27.4993	41.7756	27.4521	0.0000				
**Paxl**	34.8938	53.7417	57.1846	43.2011	40.8961	55.1692	43.6572	0.0000			
**Penc**	38.1411	28.8606	27.2594	22.3204	26.7943	28.9016	32.7680	43.5041	0.0000		
**Proc**	33.8831	32.9151	33.1340	31.7296	36.8900	32.3815	32.7072	40.8838	25.5972	0.0000	
**Vala**	42.6690	33.1038	37.1856	31.6187	27.1553	33.0821	37.8398	42.4093	21.3755	30.4514	0.0000

### 5.3 Global reactivity descriptors analysis and Fukui function comparison

**Figure 12.**
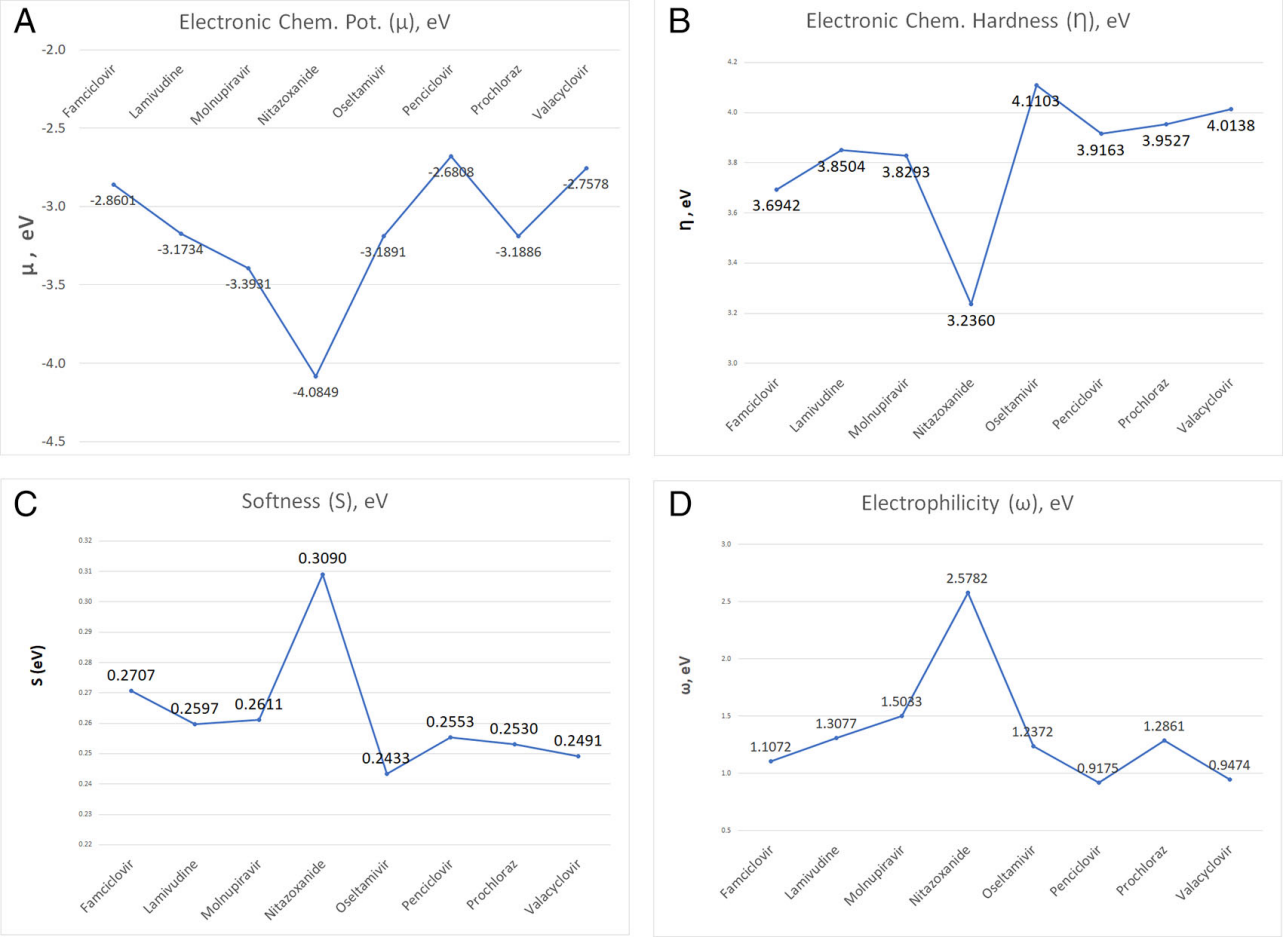
Global parameters, including electronic chemical potential (A), chemical hardness (B), global softness (C), and global electrophilicity (D) for the compounds in the study.

The investigation explored into global and local chemical reactivity descriptors through DFT computations. This part contrasts the reactivity of the ligands within the study, encompassing both overarching parameters and locally descriptive functions of reactivity. Electrophilicity values hold potential significance in stabilizing the active site of ligands engaged in non-covalent interactions. Illustrated in Figure 12 are the computed global parameters—chemical potential, chemical hardness, global softness, and global electrophilicity—offering a comparative analysis of the ligand sample’s chemical reactivity. As depicted in Figure 12, Nitazoxanide emerges as the most reactive molecule, displaying the lowest values of electronic chemical potential (μ) and chemical hardness ( *η*), alongside the highest global softness and global electrophilicity. Conversely, the remaining compounds exhibit descriptor values that display less pronounced differences. Consideration of solely global descriptors might suggest comparable reactivity. The values obtained in each case are detailed in **Table S1** in the accompanying supplementary information.

**Figure 13.**
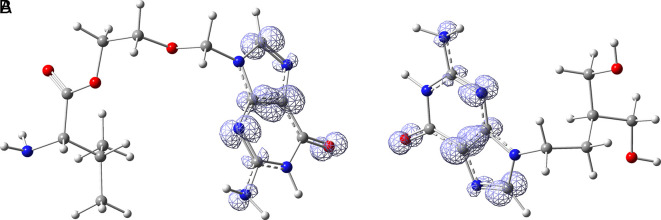
Fukui function $f^{-}(\vec{r})$ calculated under the FMO approximation $\left({\left|\text{HOMO}(\vec{r})\right|}^{2}\right)$ for the ligands A) Valacyclovir and B) Penciclovir. Isovalue was 0.008 in both cases. The figure was created using GaussView 5.0.

Since the analysis of the global parameters is limited, we will complete it with the comparison of some local descriptor functions. The electrophile and nucleophile Fukui functions (as a measure of reactivity) were then compared using the Frontier Molecular Orbital (FMO) approach. The electrophilic-nucleophilic character of the following functions also shows those molecular areas that are most likely to form charge-donating interactions (basically by charge delocalisation). These types of interactions are important and difficult to determine using docking analysis. Figure 13 shows Fukui function $f^{-}\left(\vec{r}\right)$ calculated under the FMO approximation ( ${\left|\text{HOMO}\left(\vec{r}\right)\right|}^{2}$ ) for the compounds Valacyclovir and Penciclovir (A and B respectively), it can be noted that $f^{-}\left(\vec{r}\right)$ is similar in both compounds, and in both cases, the function assigns the most nucleophilic character to the condensed rings. In **Figures S1-S8, see Underlying Data**, the $f^{-}\left(\vec{r}\right)$ functions for all ligands in the study can be seen. Comparing the ${\left|\text{HOMO}\left(\vec{r}\right)\right|}^{2}$ function from Figure 13-B with Figure 8, it can be observed that the interactions of Penciclovir with GLY 143, HIE 41, and LEU 141 obtained through docking are compatible with the $f^{-}\left(\vec{r}\right)$ function.

**Figure 14.**
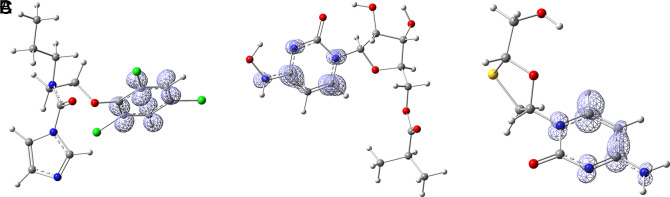
Fukui function $f^{+}(\vec{r})$ calculated under the FMO approximation $\left({\left|\text{LUMO}(\vec{r})\right|}^{2}\right)$ for the ligands A) Prochloraz, B) Molnupiravir and C) Lamivudine. The isovalue was 0.008 in all cases. The figure was created using GaussView 5.0.

Figure 14 the functions $f^{+}\left(\vec{r}\right)$ calculated under the FMO approximation ( ${\left|\text{LUMO}\left(\vec{r}\right)\right|}^{2}$ ) for compounds A) Prochloraz, B) Molnupiravir and C) Lamivudine. It can be noted that $f^{+}\left(\vec{r}\right)$ is similar in the three compounds; in all three cases, the function assigns the most nucleophilic character to various carbons in the six-carbon ring. The ring may have some ability to rotate and orient itself towards where it can form the strongest interactions. Comparing Figure 14 with Figure 2, it can be seen that $f^{+}\left(\vec{r}\right)$ is compatible with the interaction of Prochloraz with HIE 41. A similar conclusion is drawn when comparing Figure 14 with Figure 5, in this case, the interaction is formed between Molnupiravir and PHE 140. Lastly, comparing Figure 14 with Figure 10 highlights the interaction between Lamivudine and GLU 166.

**Figure 15.**
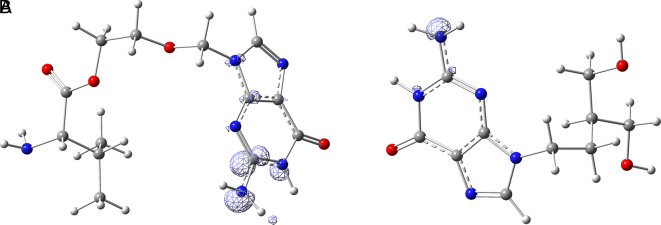
Fukui function $f^{+}(\vec{r})$ calculated under the FMO approximation $\left({\left|\text{LUMO}(\vec{r})\right|}^{2}\right)$ for the ligands A) Valacyclovir and B) Penciclovir. The isovalue was 0.008 in both cases. The figure was created using GaussView 5.0.

Figure 15 shows the functions $f^{+}\left(\vec{r}\right)$ calculated under the FMO approximation ( ${\left|\text{LUMO}\left(\vec{r}\right)\right|}^{2}$ ) for compounds **A**) Valacyclovir and **B**) Penciclovir. It can be noted that $f^{+}\left(\vec{r}\right)$ is similar in the two compounds, and in both cases, the function assigns the most nucleophilic character to the -NH2 group in the condensed rings. In Figures S1-S8, see **Underlying Data**, **mailto:https://doi.org/10.7910/DVNs/7KFPUT, Harvard Dataverse, V1**., the $f^{+}\left(\vec{r}\right)$ functions for all ligands in the study can be seen.

## 6. Conclusions

The study on ligands based on the crystal structure of SARS-CoV-2 RNA-dependent RNA polymerase with PDB code 6m71 provides valuable insights into potential drugs for COVID-19. The conclusions highlight the challenges in drug discovery for COVID-19, including virus mutability, the rapid evolution of the pandemic, lack of pre-existing therapies, complexity of the virus life cycle, drug safety, drug delivery challenges, antibody resistance, global collaboration, vaccine success, and resource limitations. Despite these challenges, the scientific community has made remarkable progress in developing vaccines and exploring therapeutic approaches.

The docking results for various ligands, including Oseltamivir, Prochloraz, Valacyclovir, Baricitinib, Molnupiravir, Penciclovir, Famciclovir, Lamivudine, and Nitazoxanide, reveal specific interactions with key residues of the SARS-CoV-2 RNA-dependent RNA polymerase. These interactions provide valuable information about the potential efficacy of these ligands in inhibiting the virus.

The global reactivity descriptors analysis, including electronic chemical potential, chemical hardness, global softness, and global electrophilicity, offers a comparative analysis of the ligands’ chemical reactivity. Nitazoxanide emerges as the most reactive molecule, displaying the lowest electronic chemical potential and chemical hardness, along with the highest global softness and global electrophilicity. The other compounds show less pronounced differences in their reactivity.

The Fukui function comparison provides additional insights into the local chemical reactivity of the ligands. For example, the comparison of Fukui functions for Valacyclovir and Penciclovir shows similar nucleophilic character in both compounds, particularly in the condensed rings.

The study is comprehensive, integrating docking results with global and local reactivity descriptors to assess the potential of various ligands as drugs for COVID-19. The information presented contributes to our understanding of the interactions between ligands and the SARS-CoV-2 RNA-dependent RNA polymerase, aiding in the development of targeted therapies.

The higher MQSM is observed between Lamivudine and Molnupiravir, indicating electronic similarity. However, the comparison between Oseltamivir and Prochloraz suggests lower electronic similarity, implying structural dissimilarity. The Coulomb operator reveals higher electronic similarity between Lamivudine and Molnupiravir, consistent with the overlap operator. The lower electronic similarity between Baricitinib and Famciclovir suggests greater structural diversity. In both cases, the electronic similarity indices using the Coulomb operator are higher than those using the overlap operator, emphasizing the electronic aspects in the molecular analysis. Further investigation into the electronic properties of the ligands is warranted. These findings enhance our understanding of the molecular properties and guide future drug discovery efforts based on both structural and electronic considerations.

## Data Availability

Harvard Dataverse: Replication data for Study of a series of ligands used as inhibitors of the SARS-CoV-2 virus. Morales-Bayuelo, Alejandro, 2023, “Replication data for Study of a series of ligands used as inhibitors of the SARS-CoV-2 virus”, https://doi.org/10.7910/DVN/7KFPUT. ^47^ Data are available under the terms of the Creative Commons Zero “No rights reserved” data waiver (CC0 1.0 Public domain dedication).
