# Supplementary material for: Study anti-viral drugs for their efficiency against multiple SARS CoV-2 drug targets within molecular docking, molecular quantum similarity, and chemical reactivity indices frameworks
Source: F1000Res. 2024 Dec 6;13:270. Originally published 2024 Apr 15. [Version 2] doi: 10.12688/f1000research.146350.2 (PMC11915800; doi:10.12688/f1000research.146350.2)
Supplement: Peer review history

# Reviewer report: Reviewer response for version 2

**DOI:** 10.5256/f1000research.174897.r368895

**Published:** 2025-03-13

**Authors:** Estari Mamidaal

**Affiliations:** 1 Kakatiya University, Warangal, Telangana, India

**General Comments:**

 The study presents an investigation into antiviral drug interactions with SARS-CoV-2 using computational methods. While the research topic is relevant and of scientific interest, there are several significant concerns regarding the study design, methodology, and data interpretation. The following points outline key issues that need to be addressed to improve the manuscript’s quality and scientific rigor.

**Major Concerns:**

1. **Study Design Appropriateness:**

The study design lacks clarity and does not adequately justify the methodology used. The selection criteria for antiviral drugs and molecular docking parameters should be clearly described to ensure reproducibility and reliability of the results.

1. **Rationale for Drug Selection:**

The manuscript does not provide a clear rationale for the selection of specific antiviral drugs tested against SARS-CoV-2. It is essential to explain why these particular drugs were chosen over others, especially when multiple antiviral compounds are available for evaluation.

1. **Source of Drug Data:**

The source or database from which the antiviral drugs were retrieved is not mentioned. It is crucial to specify whether the drugs were obtained from PubChem, DrugBank, or another reliable database to ensure data credibility and accuracy.

1. **Selection of RdRp as the Sole Target:**

The study focuses exclusively on the RNA-dependent RNA polymerase (RdRp) of SARS-CoV-2. However, the manuscript does not justify why other key viral targets, such as the main protease (Mpro) or spike protein (S-protein), were not included. A proper explanation is necessary to validate the selection of RdRp over other potential drug targets.

1. **Lack of Binding Energy Data:**

Binding energy values are critical for assessing the efficacy of antiviral drugs in docking studies. The manuscript does not provide binding energy results for the tested antiviral drugs, making it difficult to evaluate their relative effectiveness in inhibiting SARS-CoV-2 RdRp.

1. **Absence of a Standard Drug for Comparison:**

A reference or standard antiviral drug should be included to compare the docking results with experimental data or FDA-approved drugs. The absence of such a control makes it difficult to determine the reliability of the selected antiviral compounds.

 You can read these two papers and cite for clear analysis of molecular docking results:

 Swapna K, et al., 2024 (Ref 1)

 Daipule K, et al., 2020 (Ref 2)

**Conclusion:**

 The manuscript presents a relevant topic but requires significant revisions to improve its clarity, justification of methodologies, and inclusion of essential data such as drug selection criteria, binding energies, and comparison with standard drugs. Addressing these concerns will strengthen the study and enhance its contribution to the field of antiviral drug research.

**Recommendation:**

 Major Revision Required.

Is the work clearly and accurately presented and does it cite the current literature?

Yes

If applicable, is the statistical analysis and its interpretation appropriate?

Not applicable

Are all the source data underlying the results available to ensure full reproducibility?

Yes

Is the study design appropriate and is the work technically sound?

No

Are the conclusions drawn adequately supported by the results?

Yes

Are sufficient details of methods and analysis provided to allow replication by others?

Yes

Reviewer Expertise:

Computational Biology & Bioinformatics

I confirm that I have read this submission and believe that I have an appropriate level of expertise to confirm that it is of an acceptable scientific standard, however I have significant reservations, as outlined above.

## References

[1] : Identification of steroidal cardenolides from Calotropis procera as novel HIV-1 PR inhibitors: A molecular docking & molecular dynamics simulation study. *Indian J Med Res* .2024;160(1) : 10.25259/IJMR_2115_23 78-86 10.25259/IJMR_2115_23 39382500 PMC11463882

[2] : Synthesis, molecular docking simulation, and biological evaluation studies of novel amide and ether conjugates of 2,3‐diaryl‐1,3‐thiazolidin‐4‐ones. *Journal of Heterocyclic Chemistry* .2020;57(2) : 10.1002/jhet.3819 774-790 10.1002/jhet.3819

# Reviewer report: Reviewer response for version 2

**DOI:** 10.5256/f1000research.174897.r369908

**Published:** 2025-03-08

**Authors:** Taufik Muhammad Fakih

**Affiliations:** 1 Universitas Islam Bandung, Bandung, West Java, Indonesia

This paper presents a comprehensive computational study on potential COVID-19 drug candidates by integrating molecular docking, molecular quantum similarity (MQS), and chemical reactivity indices to analyze ligand interactions with SARS-CoV-2 RNA-dependent RNA polymerase (RdRp). While the study effectively identifies electronic and structural similarities among ligands, its reliance solely on in silico methods without experimental validation or molecular dynamics (MD) simulations limits its predictive power regarding real-world biological activity. The findings suggest that ligands like Lamivudine and Molnupiravir exhibit high electronic similarity, potentially enhancing their antiviral efficacy, but further experimental studies are needed to confirm their therapeutic potential. Additionally, incorporating a broader set of novel ligands and validating results through MD simulations could significantly strengthen the study's impact in antiviral drug discovery.

Is the work clearly and accurately presented and does it cite the current literature?

Yes

If applicable, is the statistical analysis and its interpretation appropriate?

Yes

Are all the source data underlying the results available to ensure full reproducibility?

Yes

Is the study design appropriate and is the work technically sound?

Yes

Are the conclusions drawn adequately supported by the results?

Yes

Are sufficient details of methods and analysis provided to allow replication by others?

Yes

Reviewer Expertise:

Computational study

I confirm that I have read this submission and believe that I have an appropriate level of expertise to confirm that it is of an acceptable scientific standard.

# Reviewer report: Reviewer response for version 2

**DOI:** 10.5256/f1000research.174897.r292820

**Published:** 2025-02-03

**Authors:** Mohnad Abdalla

**Affiliations:** 1 Shandong University, Jinan, Shandong, China

**Scope and Depth of Analysis**

 The study focuses solely on in-silico approaches, including molecular docking, molecular dynamics (MD), and QSAR studies. While these methods are valuable for preliminary investigations, the analysis lacks sufficient depth and comprehensiveness to support strong conclusions.

**Molecular Dynamics (MD) Limitations**

 The MD simulation was conducted for only 100 ns, which is insufficient to conclusively determine the stability of the protein-ligand complex. Prolonged simulations, typically in the range of 200–500 ns, are necessary to provide a more reliable insight into binding stability and interaction patterns.

**Validation Concerns**

 A critical issue with the manuscript is the absence of experimental validation. In-silico findings, while valuable, must be corroborated with laboratory studies to ensure biological relevance and reliability. Without experimental data, the conclusions remain speculative and lack practical applicability.

**Insufficient Analysis of Protein-Ligand Stability**

 The parameters assessed in the MD and QSAR studies are not sufficient to convincingly demonstrate the stability and efficacy of the protein-ligand complex. Additional analyses, such as free energy calculations and more extensive binding interaction studies, would strengthen the findings.

**Significance and Practical Relevance**

 The study does not provide a clear pathway to translate the in-silico findings into a meaningful biological or therapeutic context. This diminishes its overall impact and relevance.

**Recommendation**

 Given the above concerns, especially the lack of experimental validation and insufficient MD analysis, I recommend not indexing the manuscript in its current form. The authors are encouraged to conduct complementary laboratory experiments and provide more robust in-silico data before resubmitting to a journal.

Is the work clearly and accurately presented and does it cite the current literature?

No

If applicable, is the statistical analysis and its interpretation appropriate?

No

Are all the source data underlying the results available to ensure full reproducibility?

No

Is the study design appropriate and is the work technically sound?

No

Are the conclusions drawn adequately supported by the results?

No

Are sufficient details of methods and analysis provided to allow replication by others?

No

Reviewer Expertise:

structural biology, computational modeling, and biopharmaceutical research.

I confirm that I have read this submission and believe that I have an appropriate level of expertise to state that I do not consider it to be of an acceptable scientific standard, for reasons outlined above.

# Reviewer report: Reviewer response for version 2

**DOI:** 10.5256/f1000research.174897.r346591

**Published:** 2025-01-25

**Authors:** Sajjan Rajpoot

**Affiliations:** 1 Cancer Signaling and Microenvironment, Fox Chase Cancer Center, Philadelphia, Pennsylvania, USA

I had provided a point wise suggestions and comments in the last version for each section of the manuscript. I do not see any changes made to those comments. Neither it has been corrected. Also, there is no response sheet provided by authors for the comments. Minor changes made as mentioned in this version are not substantially improve manuscript quality and not related to any comments provided after reviewing first version of the manuscript. To my best understanding, the manuscript is not fit for indexing in its current form.

 Thanks

Is the work clearly and accurately presented and does it cite the current literature?

Partly

If applicable, is the statistical analysis and its interpretation appropriate?

Yes

Are all the source data underlying the results available to ensure full reproducibility?

Yes

Is the study design appropriate and is the work technically sound?

Yes

Are the conclusions drawn adequately supported by the results?

Partly

Are sufficient details of methods and analysis provided to allow replication by others?

Yes

Reviewer Expertise:

Inflammation and Immunity, Drug Designing, Cancer Immunotherapy, Computational Biology.

I confirm that I have read this submission and believe that I have an appropriate level of expertise to state that I do not consider it to be of an acceptable scientific standard, for reasons outlined above.

# Reviewer report: Reviewer response for version 1

**DOI:** 10.5256/f1000research.160428.r280574

**Published:** 2024-07-23

**Authors:** Sobia Ahsan Halim

**Affiliations:** 1 Natural and Medical Sciences Research Center, University of Nizwa, Birkat Al-Mouz, Nizwa, Oman

The manuscript entitled "Study anti-viral drugs for their efficiency against multiple SARS CoV-2 drug targets within molecular docking, molecular quantum similarity, and chemical reactivity indices frameworks" by Alejandro et al., is a significant piece of work, well written and well presented. 

 In my views it should be indexed after minor revision.

 My comments are:

 The in-silico data should be validated by in-vitro test.

 The limitation of this study should be added in the end of the manuscript.

 The future recommendation of the work should be given  in the manuscript.

Is the work clearly and accurately presented and does it cite the current literature?

Yes

If applicable, is the statistical analysis and its interpretation appropriate?

Yes

Are all the source data underlying the results available to ensure full reproducibility?

Yes

Is the study design appropriate and is the work technically sound?

Yes

Are the conclusions drawn adequately supported by the results?

Yes

Are sufficient details of methods and analysis provided to allow replication by others?

Yes

Reviewer Expertise:

Computational drug design

I confirm that I have read this submission and believe that I have an appropriate level of expertise to confirm that it is of an acceptable scientific standard, however I have significant reservations, as outlined above.

# Response

**Published:** 2024-12-12

**Authors:** Alejandro Morales-Bayuelo

**Affiliations:** Universidad del Sinú, Colombia

All the revision we made according to the reviewer.

 ​​​​​​​Best Regards

# Reviewer report: Reviewer response for version 1

**DOI:** 10.5256/f1000research.160428.r292812

**Published:** 2024-07-13

**Authors:** Sajjan Rajpoot

**Affiliations:** 1 Cancer Signaling and Microenvironment, Fox Chase Cancer Center, Philadelphia, Pennsylvania, USA

The manuscript discussed the SARS-CoV-2 RdRp as the drug target for COVID-19 and attempted to study a set of anti-viral drugs including Oseltamivir, Prochloraz, Valacyclovir, Baricitinib, Molnupiravir, Penciclovir, Famciclovir, Lamivudine, and Nitazoxanide to highlight their different binding pattern with RdRp through molecular docking, and performed the drug structures comparison through molecular quantum similarity measure (MQSM) and their chemical reactivity and Fukui function comparison using multiple parameters. The manuscript suggest that ligand parameters are crucial in drug designing and these parameters of anti-viral drug including structural basis and their interaction pattern gives an insight to be considered for future drug designing.

 The effort of the authors is appreciated. Manuscript has valuable information for the readers. However, the following are the concerns and my opinion which is further suggested for authors to implement.

 Major

1. Title:
  1. The tile of the manuscript needs revision. It has mentioned multiple SARS-CoV-2 drug targets which is confusing for readers. Do authors mean multiple target sites within RdRp or multiple target proteins/enzymes of SARS-CoV-2? The title can also include “SARS-CoV-2 RdRp” to be specific as target protein.

1. Abstract:
  1. The Abstract needs revision. Either write the abstract as a single paragraph running text mentioning all the key sections of the study or structure well in category like background, methods, results and discussion and conclusion. Please include the key results of the manuscript and conclusive statement at the end.  
  2. Spell out all the abbreviations used in abstract.

1. Introduction: Overall, the introduction needs re-organization.
  1. Cite more recent and relevant references on COVID-19 epidemiology, symptoms in first paragraph.
  2. Authors should briefly include structural introduction for SARS-CoV-2 such as genome size, their key structural and non-structural proteins and further emphasize on their target protein RdRp in detail (such as its protein size, domains, active site, etc.) and why it is important to target.
  3. SRAS-CoV-2 RdRp has been studied well in recent and many in-silico studies are performed including molecular docking and molecular dynamic simulation. Authors should cite some previous studies and write how their study differ from it.
  4. Third paragraph, please cite few references with drugs under investigation.
  5. In few lines, summarize the study plan, what you have performed and what question this study is answering at the end of the introduction as closing remark.
  6. Overall, include more citations for each paragraph in introduction.

1. Methods:
  1. Section 2.1-Please add the hyperlink for PDB ID 6m71 next to it for easy access.
  2. Section 2.1- Please mention the total number of compounds used in the study and list out all the compounds either in main manuscript or a table/excel sheet in supplementary data. 

1. Results and Discussion
  1. Overall, the results in section 5.1 are not well written and discussed. Authors needs to focus more on their outcomes-based text in the result and discussion section. Authors should give the rational for molecular docking study in the beginning and conclude their docking result with their finding before moving to next study in section 3.1.
  2. As mentioned above, write how many compounds were total docked with RdRp and what analysis led to identify the best compounds and binding pose of the complex? Are the representative figures (Fig 1-11) being the best binding ligands? Are interacting residues of RdRp with ligands mentioned in manuscript are crucial in limiting the RdRp function? Include these in your results.
  3. Section 5.2- please co-relate how the outcome of the molecular docking complement with your subsequent study in the MSQM section. Does the similarity analysis of ligands co-relate with the binding pose and binding strength/affinity of the ligands within the RdRp?  
  4. For Fig 1-11, it has two sub-figures for each, as 3D and 2D model. Authors should label them as A, B.. or i, ii.. as per journal format.
  5. For Fig 1-11, besides the main figure title, add well explained figure legend for each sub-section along with color codes, the visualization tool and its version used for representative figure preparation.
  6. For Fig 1-11, authors may also add the color label obtained from their docking visualization tool defining the type of interactions between ligand and target protein.
  7. Authors have mentioned about the binding energy (IFDScore) and docking energy (GScore) in methods. How does these energies were useful in your study to understand the binding strength of the ligands and comparison between them besides the interaction with RdRp residues. Please write in your results. Also, the energies for the docking complex can be summarized and presented in a table for better understanding. Also, this will help to understand the binding strength in comparison to structure similarity.
  8. In continuation to above comment (f), considering Section 2.2 and section 5.1, authors have mentioned about the molecular dynamics calculation over 30s to predict the best poses. Please elaborate on that. Does it mean the molecular dynamics simulation performed on the RdRp and drug complex? Also, the same should be explained in result section. Please write the result of the calculation that how it supported in finding the best pose.
  9. Section 5.3- Fig.12, The graph needs improvement. If possible, the authors are suggested to use Prism-GraphPad/Origin or other similar software for representative graph preparation. Overall, please make sure the graphs are uniform in terms of representation-like the x-axis and y-axis font size should be same. Fig.12A, the x-axis label (drugs name) is below the top x-axis line, please correct. Add axis lines for each graph, remove the inside grid lines. Increase the font size, it’s not clearly visible.
  10. Section 5.3- Are there any standard reference values for electronic chemical potential (A), chemical hardness (B), global softness (C), and global electrophilicity to define the significance of the global parameters of the ligand? If any, please provide and add to the manuscript and in graph legend.

1. Conclusion
  1. I didn’t understand the conclusion very well. The conclusion is dispersed. I believe an overall conclusion at the end is missing. As per the findings, summarize that which of the key ligand(s) can be considered better including all the parameters of the study.
  2. Authors should also write the limitations of their study and future-plan to overcome that.

Minor

1. All abbreviation should be spelled out at first use. Spell out SARS-CoV-2 in the Introduction section.
2. Section 5.1- Please correct “serie of ligands” to “series of ligands”.
3. Make sure all the journal article guidelines are followed.

The authors have done well. Further, I believe the above-mentioned comments will be constructive to add more quality to the manuscript and information for readers.

Is the work clearly and accurately presented and does it cite the current literature?

Partly

If applicable, is the statistical analysis and its interpretation appropriate?

Yes

Are all the source data underlying the results available to ensure full reproducibility?

Yes

Is the study design appropriate and is the work technically sound?

Yes

Are the conclusions drawn adequately supported by the results?

Partly

Are sufficient details of methods and analysis provided to allow replication by others?

Yes

Reviewer Expertise:

Inflammation and Immunity, Drug Designing, Cancer Immunotherapy, Computational Biology.

I confirm that I have read this submission and believe that I have an appropriate level of expertise to confirm that it is of an acceptable scientific standard, however I have significant reservations, as outlined above.
